# Shredding artifacts: extracting brain activity in EEG from extreme artifacts during skateboarding using ASR and ICA

**DOI:** 10.3389/fnrgo.2024.1358660

**Published:** 2024-06-26

**Authors:** Daniel E. Callan, Juan Jesus Torre–Tresols, Jamie Laguerta, Shin Ishii

**Affiliations:** ^1^Brain Information Communication Research Laboratory, Advanced Telecommunications Research Institute International, Kyoto, Japan; ^2^Institut Supérieur de l'Aéronautique et de l'Espace, University of Toulouse, Toulouse, France; ^3^Department of Integrated Engineering, University of British Columbia, Vancouver, BC, Canada; ^4^Graduate School of Informatics, Kyoto University, Kyoto, Japan

**Keywords:** EEG, auditory evoked potential, artifact, machine learning, ASR, Independent Component Analysis, EEGLAB Pipeline, sports

## Abstract

**Introduction:**

To understand brain function in natural real-world settings, it is crucial to acquire brain activity data in noisy environments with diverse artifacts. Electroencephalography (EEG), while susceptible to environmental and physiological artifacts, can be cleaned using advanced signal processing techniques like Artifact Subspace Reconstruction (ASR) and Independent Component Analysis (ICA). This study aims to demonstrate that ASR and ICA can effectively extract brain activity from the substantial artifacts occurring while skateboarding on a half-pipe ramp.

**Methods:**

A dual-task paradigm was used, where subjects were presented with auditory stimuli during skateboarding and rest conditions. The effectiveness of ASR and ICA in cleaning artifacts was evaluated using a support vector machine to classify the presence or absence of a sound stimulus in single-trial EEG data. The study evaluated the effectiveness of ASR and ICA in artifact cleaning using five different pipelines: (1) Minimal cleaning (bandpass filtering), (2) ASR only, (3) ICA only, (4) ICA followed by ASR (ICAASR), and (5) ASR preceding ICA (ASRICA). Three skateboarders participated in the experiment.

**Results:**

Results showed that all ICA-containing pipelines, especially ASRICA (69%, 68%, 63%), outperformed minimal cleaning (55%, 52%, 50%) in single-trial classification during skateboarding. The ASRICA pipeline performed significantly better than other pipelines containing ICA for two of the three subjects, with no other pipeline performing better than ASRICA. The superior performance of ASRICA likely results from ASR removing non-stationary artifacts, enhancing ICA decomposition. Evidenced by ASRICA identifying more brain components via ICLabel than ICA alone or ICAASR for all subjects. For the rest condition, with fewer artifacts, the ASRICA pipeline (71%, 82%, 75%) showed slight improvement over minimal cleaning (73%, 70%, 72%), performing significantly better for two subjects.

**Discussion:**

This study demonstrates that ASRICA can effectively clean artifacts to extract single-trial brain activity during skateboarding. These findings affirm the feasibility of recording brain activity during physically demanding tasks involving substantial body movement, laying the groundwork for future research into the neural processes governing complex and coordinated body movements.

## Introduction

It was once widely maintained that successful brain recording using head mounted EEG was only possible under very strict laboratory conditions that included recording while sitting still with very little head and body movement within an electrically shielded and acoustically attenuated room (Luck, 2014). With the advent of more portable EEG hardware and the development of sophisticated artifact cleaning signal processing methods, neuroergonomic research has been conducted during such real-world activities as walking (Gwin et al., 2010; Kline et al., 2015; Jacobsen et al., 2020), running (Gwin et al., 2010; Gorjan et al., 2022), playing the guitar (Muller et al., 2013; Sasaki et al., 2019); cycling (Zink et al., 2016; Scanlon et al., 2019), driving in cars (Zhang et al., 2015), and flying in airplanes (Callan et al., 2015, 2018).

The goal of this study is to objectively assess the extent to which brain related electroencephalography EEG can be successfully recorded during sports activity requiring considerable body movement. In this case we are particularly interested in skateboarding on a half-pipe ramp. This research is important in establishing EEG based methodology that can be used for neuroergonomic investigation of brain function in natural real-world settings. To better understand how the brain functions it is critically important to move outside of the laboratory setting and investigate it in natural real-world environments and situations (Parasuraman and Rizzo, 2008; Makeig et al., 2009; Gramann et al., 2014, 2021; Ladouce et al., 2017; Callan and Dehais, 2018; Dehais et al., 2020; Fairclough and Lotte, 2020).

ASR and ICA are two artifact cleaning pre-processing methods that have been shown to improve brain related EEG signal in numerous experiments (Mullen et al., 2013; Callan et al., 2015; Kothe and Jung, 2015; Chang et al., 2020; Gorjan et al., 2022). Human movement related artifacts (in the case of gait) are complex and difficult to detect and cannot be removed by simple artifact cleaning methods such as band-pass filtering (Kline et al., 2015; Gorjan et al., 2022). ASR (Artifact Subspace Reconstruction) is an automatic method designed for the removal of non-stationary components from multi-channel EEG data (Mullen et al., 2013; Kothe and Jung, 2015; Chang et al., 2020; Gorjan et al., 2022). The technique involves employing a sliding window on the EEG data and applying PCA (Principal Component Analysis) decomposition to each window. ASR automatically detects and utilizes clean segments of data to establish thresholds for rejecting components with large variance. Subsequently, the channel data is reconstructed from the components that are retained (Chang et al., 2020; Gorjan et al., 2022). Independent Component Analysis (ICA) in the context of EEG refers to a signal processing technique used to automatically separate a multichannel EEG recording into “maximally” temporally independent and spatially stationary sources each representing a different underlying physiological activity and/or artifact (Makeig et al., 1996, 2004; Onton and Makeig, 2006; Artoni et al., 2018; Gorjan et al., 2022). This allows for the isolation and identification of various brain-related and non-brain-related signals, such as physiological artifacts (e.g., eye movement and muscle activity), motion artifacts, and specific brain related activities, contributing to the overall EEG recording. Combining ASR and ICA is thought to improve overall artifact cleaning of the EEG signal for extraction of brain related activity (Gorjan et al., 2022). This is partly a result of ASR being able to remove non-stationary transient artifacts from the EEG data. Non-stationary artifacts in EEG can arise from such things as impedance changes due to headset motions as well as transients caused by abrupt movement, impact, or other physiological or environmental factors (Hsu et al., 2016; Chang et al., 2020). These types of transient artifacts are expected in real-world conditions characteristic of sports in general. Non-stationary transient artifacts are often problematic for ICA decomposition because they violate ICA's assumption of spatiotemporal stationarity of the data making it difficult for decomposition to converge which may cause greater mixing of underlying brain sources and artifacts into single components. As a consequence of ASR's preceding removal of transient non-stationary artifacts from the EEG data, there is an enhancement in the effectiveness of ICA decomposition (Chang et al., 2020). It should be noted that there are variants of ICA that are better able to deal with non-stationarities in the EEG data such as ORICA (Hsu et al., 2016) and AMICA (Palmer et al., 2007). The combination of ASR and ICA has been used successfully to extract relevant brain activity in many EEG studies (Callan et al., 2015, 2018, 2023; Sasaki et al., 2019; Gorjan et al., 2022).

Before utilizing EEG for a new experimental paradigm (in this case skateboarding on a half-pipe ramp), it is important to validate artifact cleaning methods using a dual task paradigm for which characteristic brain activity is known while concurrently performing the new task that lacks ground truth for distinguishing between noisy and clean samples. The dual-task paradigm has been used in several studies to validate that extraction of brain related activity is possible in real-world situations including walking/running (Gwin et al., 2010), cycling (Zink et al., 2016; Scanlon et al., 2019), electric skateboarding (Robles et al., 2021), and flying airplanes (Callan et al., 2015). All of these studies used evaluation of the event-related potential to auditory or visual stimuli for validating the presence of brain related EEG activity. The method employed in the Callan et al. (2015) study is unique in that it employed the use of machine learning to evaluate EEG cleaning performance at the single trial level unlike the other studies that required averaging over multiple trials.

The Callan et al. (2015) study demonstrated that by utilizing ASR and ICA to clean the EEG data that single trial auditory responses could be classified (presence or absence of an auditory stimuli) using machine learning techniques while piloting an open cockpit biplane (Callan et al., 2015). The classification performance while piloting on the single trial EEG data was greatly improved by utilizing ASR and ICA (77.3%) compared to minimal artifact cleaning using band-pass filtering (66.1%). The control condition, in which the subject sat in the biplane on the ground with the engine and avionics off, showed that classification performance was relatively good both with minimal artifact cleaning (77.4%) as well as with artifact cleaning using ASR and ICA (78.5%) (Callan et al., 2015). The results of the Callan et al. (2015) study demonstrate that it is possible (at least for the subject under investigation and likely extrapolates to others as well) to extract brain related EEG activity at a single trial (or continuous) level by utilizing ASR and ICA artifact cleaning methods.

In this study, we utilize this same dual task paradigm (Callan et al., 2015) to determine if it is possible to extract brain related single trial EEG during skateboarding on a half-pipe ramp by using machine learning methods to assess classification performance of detecting the presence vs. the absence of a sound stimuli at the single trial level. Skateboarding is likely to be particularly challenging for artifact cleaning as there is likely to be considerable artifacts resulting from motion of the body, muscle activity, and impact of the board on the ramp (resulting in non-stationary transient artifacts). The experiment consists of two task conditions skateboarding on the ramp and resting (sitting still) while listening to auditory stimuli. Five different artifact cleaning pipeline conditions were tested (minimal cleaning, ASR alone, ICA alone, ICA with ASR after, and ASR before ICA) to assess the contribution of the use of ASR and ICA alone as well as the ordering of the processing steps. Based on the ability of ASR to remove non stationarities in EEG that facilitates ICA decomposition, it is predicted that ASR prior to ICA should show the best artifact cleaning performance.

## Methods

### Subjects

Three male subjects with expert level skateboarding skill participated in this experiment. The ages of the subjects were as follows: Subject 1 = 23 years; Subject 2 = 25 years; Subject 3 = 29 years. All of the subjects reported having normal vision and normal hearing. Two of the subjects are right-handed (as determined by Edinburgh handedness questionnaire) and one of the subjects (Subject 1) reported left-handed for writing, drawing, and scissors, but right dominance for throwing and kicking during skateboarding. The Subjects have 12, 17, and 11 years of experience respectively for half pipe ramp skateboarding. The experimental procedures were approved by the ATR Human Subject Review Committee (ethics approval number 158) and were carried out in accordance with the principles expressed in the WMA Declaration of Helsinki. The confidentiality rights of all participants were observed.

### Experimental procedure

The experiment consisted of an auditory listening task and a skateboarding task. The auditory task consisted of listening to two chirp sounds (sweep up from 2 to 4 kHz and sweep down from 4 to 2 kHz with a 0.1 s duration). The auditory task was to passively listen to the chirp sounds. The sounds were played at the maximum comfortable audio level each subject reported they could tolerate for the duration of the entire experiment which lasted around 40 min. The audio stimuli were presented to the subjects using the low latency EPOS GTW 270 Bluetooth earbuds together with the aptX Bluetooth transmitter. Two cables were connected from the computer's audio output. One cable was linked to the low-latency aptX Bluetooth transmitter, while the other was connected to the Cognionics Trigger Box (sending trigger marker to EEG recording). The trigger box's threshold was adjusted to detect the initiation of audio stimuli. There was an ~45 ms delay in the presentation of the sound stimuli to the subject that was not adjusted in this experiment. See Callan et al., 2015, 2018, 2023 for experiments using similar stimuli and methods.

**Figure 1:**
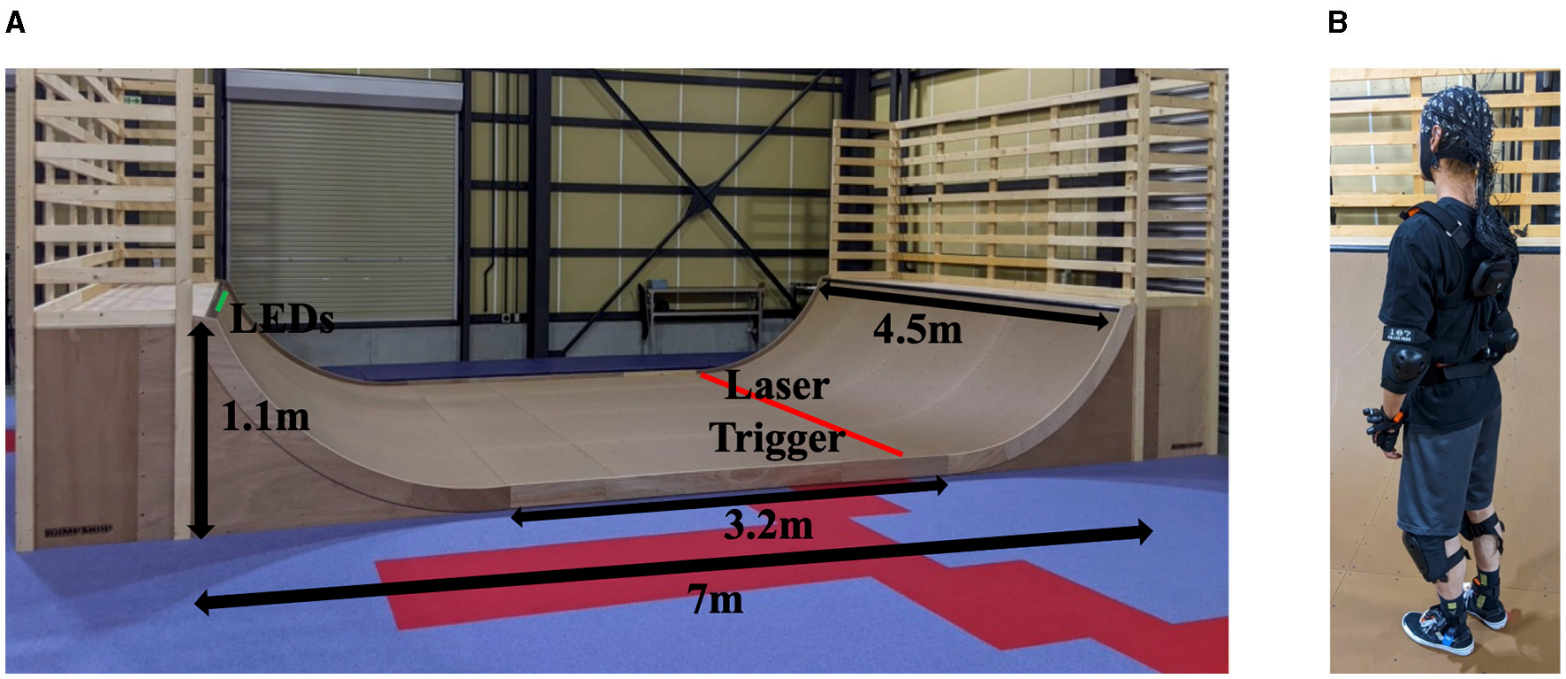
**(A)** Skateboard Half-pipe ramp at ATR robot laboratory. The dimensions of the ramp and position of the laser trigger and LED light strip are also given in the figure. **(B)** The CGX Mobile-72 high density EEG system on the skateboarder. The breakout box with the wireless module is shown attached to a harness on the back of the skateboarder. A helmet is worn over the EEG cap during the entire experiment.

The skateboarding task consisted of performing pumping (up and down motion of the body on the skateboard to accelerate up the ramp) and frontside (turn made with front facing the ramp) and backside (turn made with back facing the ramp) kickturn maneuvers on a half-pipe skateboard ramp (Skateboard Ramp at ATR Robot Laboratory, Figure 1A). The direction and location on the ramp of the kickturn (to the right or left) was determined by a bank of LED lights. A red light signaled to turn to the right, whereas, a green light signaled to turn to the left. The onset of the LED light was triggered when the skateboarder passed through a laser at the opposite end of the skateboard ramp. The kickturns occurred on the LED side of the ramp and the pumping occurred on the opposite side of the ramp.

The experiment switched between sets of rest and skateboarding during which the auditory task was carried out. Each set lasted ~100 s. For the skateboarding task each set consisted of continuously skateboarding on the ramp completing 20 kickturns on the LED side of the ramp (equal number of frontside and backside kickturns to the left and right pseudo randomly determined) and 20 pumping maneuvers on the opposite side of the ramp. For the auditory task the two chirp sounds were presented 20 times each with an interstimulus interval of 1.5–2 s randomly determined. The presentation of the two chirp sounds was pseudo randomly determined such that the number of stimuli was equal. In total there were 10 resting sets and 10 skateboarding sets for a total of 400 sound presentations for each the resting and skateboarding conditions throughout the total 40-min experimental duration.

### EEG data collection and preprocessing

The EEG data was recorded using the Cognionics 72 channel active electrode wireless mobile EEG system (CGX Mobile-72, 500 Hz sampling rate, 24-bit simultaneous sampling analog-to-digital converters, Bluetooth Wireless Range: 10 m). The CGX Mobile-72 was custom made without the electrode holders so that it was thin enough to wear a skateboard helmet on top of it. The breakout box with the wireless module was worn on a harness on the back (see Figure 1B). The EEG data was recorded continuously for two ~20-min sessions each composing of five resting and five skateboarding sets. The following pipeline, employing the EEGLAB toolbox (Delorme and Makeig, 2004), was utilized for the preprocessing of the EEG data (for similar pipelines also see Bigdely-Shamlo et al., 2015; Callan et al., 2015, 2018, 2023; Sasaki et al., 2019).

### EEG processing stages

1. The raw EEG data underwent band-pass filtering within the 3–100 Hz range utilizing a Hamming windowed Sinc FIR filter. The two sessions were filtered separately then combined for all further analyses. A somewhat high high-pass filter was selected based on what has worked best in other experiments we conducted in mobile environments (Callan et al., 2018, 2023) in terms of ICA decomposition but no systematic analysis was conducted to confirm this. The study by Klug and Gramann (2021) suggests that higher high-pass filters show better ICA decomposition in mobile environments with higher numbers of channels requiring a higher cutoff frequency (recommended 1.5–2 Hz). However, Tanner et al. (2015) suggests that high-pass filters above 0.3 Hz may produce artifactual effects. pop_eegfiltnew.
2. The Cleanline EEGLAB toolbox (default settings) was employed to eliminate line noise at 60 Hz. pop_cleanline.
3. The automatic rejection of channels relied on a weak correlation with a robust estimate derived from other channels (0.8 threshold) and a flat line duration criterion of 5 s (EEGLAB program Clean Rawdata). pop_clean_rawdata.
4. Non-stationary high-variance signals were removed from the EEG using Artifact Subspace Reconstruction (ASR) (using Euclidean distance) [refer to Mullen et al. (2013) and Kothe and Jung (2015)]. The removal criteria included a standard deviation cutoff of 20 for burst removal. No time windows were removed for data that could not be repaired. pop_clean_rawdata.
5. The rejected channels were interpolated using the “spherical” method (EEGLAB). pop_interp.
6. Common average referencing of channels was performed (EEGLAB). To maintain the original rank and prevent a loss of one rank due to average referencing, an additional channel with all zeros was introduced. pop_reref.
7. Independent Component Analysis (ICA) with Principal Component Analysis (PCA) reduction was carried out on the dataset (EEGLAB extended “infomax” ICA program). The dimension of the PCA reduction was determined by the number of rejected channels and was carried out prior to ICA decomposition. PCA reduction was carried out to account for the loss of rank resulting from channel removal and then channel interpolation (failure to account for the loss of rank may degrade ICA decomposition). However, see Artoni et al. (2018) which suggests that PCA removal before ICA may also cause degradation of ICA quality. Interpolation of electrodes is not possible after ICA, therefore, we opted to use interpolation followed by average referencing before ICA with PCA reduction. pop_runica.
8. In this study, ICLabel version 1.4 (Current version at time of analysis was conducted) was employed to distinguish between brain-related and artifact-related independent components (ICs). ICLabel is a toolbox designed for the automated classification of ICs into seven categories: Brain, Muscle, Eye, Heart, Line Noise, Channel Noise, and Other, as introduced by Pion-Tonachini et al. (2019). The categorization process in ICLabel utilizes IC topomaps, power spectral density within the 3–100 Hz range, and equivalent current dipole information. Our study specifically considered ICs labeled as “Brain” when the percentage of “Brain” categorization exceeded 50%. It is possible that some potential brain components may have been missed by including the other category as artifacts. pop_iclabel.
9. Only independent components (ICs) labeled as “brain” were retained, and all other ICs were excluded from the dataset. Subsequently, solely brain-related ICs were projected onto the EEG electrodes.

Five distinct data cleaning approaches were employed to assess their efficacy in extracting artifacts during skateboarding. It is important to note that all pipelines used the same number of channels (72 total), using the same interpolation and average referencing methods. Additionally, none of the data was removed for any of the analyses and therefore the exact same trials were assessed for all the cleaning approaches. The objective was to discern from single-trial auditory responses whether presence or absence of an auditory sound stimuli could be classified (using machine learning methods, see below) from the channel-level EEG data. These five cleaning pipelines are as follows:

1. Minimal: incorporated the stages of the pipeline up to and including the common average referencing of the channels, excluding the Artifact Subspace Reconstruction (ASR) step. (stages 1, 2, 3, 5, 6).
2. ASR: incorporated stages (1, 2, 3, 4, 5, 6).
3. ICA: incorporated stages (1, 2, 3, 5, 6, 7, 8, 9).
4. ICAASR: incorporated stages (1, 2, 3, 5, 6, 7, 8, 9, 4).
5. ASRICA: incorporated all stages of the pipeline in the following order (1, 2, 3, 4, 5, 6, 7, 8, 9).

### Classification analysis

A two-class classification problem was analyzed, with classes sound stimulus present and sound stimulus absent. All classification analyses used a support vector machine SVM with a radial basis function kernel and were carried out in Python, using the open-source library scikit-learn. The kernel and other SVC parameters are all the scikit-learn defaults. Since the goal of this study is to show the effect of pre-processing and that brain activity is recoverable under “extreme artifacts,” we thought it would be best to use a simple, out-of-the-box model, without optimizing classification parameters. Optimizing for classification parameters could lead to overfitting.

In order to extract the epochs for each class, each EEG trigger for sound presentation was used as an onset reference time point. For each trigger, an epoch from onset to 750 ms after was extracted to create a sound trial, while another epoch from −750 ms to onset was extracted to create a sound absent trial. The features employed for classification via SVM included the amplitude of the 375 samples within the 750 ms trial time period for all 72 channels. Sample amplitude was also used for features in the Callan et al. (2015) study (discussed above). However, a different machine learning algorithm and time period was used in that study. The 750 ms time period for trials was selected as it is the longest duration segment we can have without potential overlap given the random interstimulus interval from 1.5 to 2 s. Using a longer time segment allows for better assessment of the potential artifacts arising from the skateboarding task. Classification was performed using a 20-fold stratified shuffle split cross-validation (see the following for more details; https://scikit-learn.org/stable/modules/cross_validation.html#stratified-shuffle-split), where the test size of each fold was set to a 20% of the total number of trials. The same random seed was used for SVM cross-validation for all the artifact cleaning pipelines. Therefore, all the artifact cleaning pipelines had the same train/test trials across the 20-fold cross-validation. This allows for performance of the five artifact cleaning pipelines to be directly compared. The total number of trials for each subject were as follows: Subject 1 = 780 trials (397 rest and 383 skateboard), Subject 2 = 749 trials (370 rest and 379 skateboard), Subject 3 = 740 trials (369 rest and 371 skateboard). The dropout in some of the trials was a result of machine failures with the trigger delivery system.

The analysis was done separately for two distinct experimental conditions: rest and skateboarding. In order to test the contribution of the ASR and ICA to the quality of the resulting signal, we carried out the SVM classification analysis using the five artifact cleaning pipelines: minimal, ASR, ICA, ICAASR, and ASRICA. Classification accuracy for distinguishing sound from silent trials for each artifact cleaning pipelines were assessed. The proposed evaluation metric has the advantage of being able to train and test on the same trials, using the same interpolated channels for all cleaning pipelines, and can assess single trial performance. Metrics used in other studies such as using ICLabel decomposition of brain components (Pion-Tonachini et al., 2019; Chang et al., 2020) will not work for all pipelines such as minimal cleaning and ASR alone. Many other studies use averaged evoked potential data in their metric of assessing performance (Gwin et al., 2010; Zink et al., 2016; Scanlon et al., 2019; Robles et al., 2021; Delorme, 2023). Our metric, that works over single trials and does not reject any trials, is more valid in terms of neuroergonomic applications that often use machine learning methods.

## Results

The results of the EEG channel rejection, as well as the number of independent components and the number of brain components for ICA (without ASR before and with ASR before) are given for each subject in Table 1. Only a few bad channels were removed for each of the subjects (4, 2, and 1 channel(s) respectively). Utilizing ASR before ICA resulted in ICLabel identifying more brain components for all of the subjects (see Table 1).

**Table 1:** Number of Channels and ICs for the three subjects.

	**Number channels removed**	**Names of removed channels**	**Number of ICs**	**Number of brain ICs w/o ASR before ICA (ICA) (ICAASR)**	**Number of brain ICs with ASR before ICA (ASRICA)**
Sub1	4	TP7, TP8, P9, PO9	68	8	14
Sub2	2	P9, 010	70	2	11
Sub3	1	P9	71	5	8

ICLabel was used to identify brain components (threshold >0.5). IC, independent component.

The results of the classification performance for the rest and skateboarding conditions for all five artifact cleaning pipelines are given for the three subjects in Table 2. To assess whether SVM single trial classification performance for detecting presence or absence of sound was above chance (0.5) a *t*-test was conducted across the results of the 20-fold cross-validation test set results. Multiple comparisons across the 2 × 5 conditions (task × pipeline) were corrected for using the Benjamini–Hochberg correction of the False Discovery Rate FDR for *p* < 0.05 (Matlab Multiple Testing Toolbox v1.1.0). The effect size was measured using Hedge's *g*1 that is appropriate for use with one-sample *t*-tests (Matlab Toolbox: Measures of Effect Size). Values range from –inf to +inf with effect size considered as follows: small ±0.2, medium ±0.5, large ±0.8. For all three subjects (with the exception of the minimal cleaning pipeline for subject 2), all the artifact cleaning pipelines showed greater than chance performance for both rest and skateboarding conditions. The classification results in terms of signal detection theory are also given in Table 2. Hits are defined as trials that contained sound stimuli that were classified as such. False alarms are defined as trials that were absent of sound stimuli that were none the less classified as containing a sound stimulus. Sensitivity of the classifier can be measured by d′ which takes into account both the hit rate and the false alarm rate (higher d′ values are better).

**Table 2:** Classification performance for the three subjects for each of the pipeline conditions.

	**Rest Min**	**Rest ASR**	**Rest ICA**	**Rest ICAASR**	**Rest ASRICA**	**SB Min**	**SB ASR**	**SB ICA**	**SB ICAASR**	**SB ASRICA**
**Subject 1**										
M	0.728	0.719	0.681	0.683	0.705	0.547	0.676	0.669	0.668	0.691
SE	0.008	0.008	0.007	0.007	0.006	0.007	0.007	0.007	0.007	0.007
CI	0.712–0.745	0.701–0.737	0.666–0.696	0.668–0.697	0.692–0.718	0.533–0.562	0.661–0.690	0.656–0.683	0.654–0.682	0.676–0.706
T	28.81^*^	25.97^*^	25.57^*^	26.21^*^	33.05^*^	6.77^*^	24.78^*^	25.95^*^	24.81^*^	25.60^*^
ES	6.44	5.81	5.72	5.86	7.39	1.51	5.54	5.80	5.55	5.72
HR	0.757	0.759	0.701	0.703	0.727	0.817	0.685	0.664	0.662	0.675
FAR	0.300	0.321	0.339	0.337	0.317	0.722	0.334	0.325	0.326	0.292
d′	1.220	1.167	0.943	0.952	1.080	0.315	0.911	0.875	0.868	1.000
**Subject 2**										
M	0.696	0.697	0.760	0.760	0.819	0.519	0.539	0.619	0.588	0.680
SE	0.009	0.009	0.008	0.008	0.007	0.009	0.011	0.009	0.008	0.005
CI	0.677–0.715	0.679–0.714	0.741–0.777	0.741–0.777	0.805–0.833	0.500–0.537	0.515–0.563	0.601–0.638	0.572–0.605	0.671–0.691
T	21.83^*^	23.05^*^	30.56^*^	30.56^*^	47.00^*^	2.14^*^	3.44^*^	13.54^*^	11.05^*^	38.79^*^
ES	4.88	5.15	6.83	6.83	10.51	0.48	0.77	3.03	2.47	8.67
HR	0.672	0.676	0.745	0.745	0.830	0.579	0.569	0.630	0.572	0.669
FAR	0.279	0.283	0.227	0.227	0.193	0.541	0.491	0.391	0.395	0.307
d′	1.030	1.031	1.408	1.408	1.824	0.951	0.197	0.608	0.448	0.941
**Subject 3**										
M	0.724	0.722	0.708	0.712	0.746	0.498	0.569	0.638	0.639	0.631
SE	0.008	0.007	0.007	0.008	0.007	0.002	0.008	0.008	0.008	0.008
CI	0.709–0.740	0.708–0.738	0.693–0.724	0.696–0.728	0.732–0.760	0.495–0.502	0.553–0.587	0.622–0.653	0.623–0.655	0.614–0.647
T	30.54^*^	30.58^*^	28.52^*^	27.48^*^	37.15^*^	−0.99	8.78^*^	18.32^*^	18.47^*^	16.57^*^
ES	6.83	6.84	6.38	6.14	8.31	−0.22	1.96	4.10	4.13	3.71
HR	0.722	0.712	0.674	0.675	0.726	0.542	0.555	0.617	0.616	0.611
FAR	0.273	0.268	0.257	0.251	0.234	0.545	0.417	0.342	0.338	0.349
d′	1.192	1.180	1.103	1.124	1.326	−0.008	0.349	0.705	0.714	0.669

M, mean accuracy for the 20 folds (correct responses for sound trials and silent trials divided by the total number of trials). T-statistic that performance on the 20 folds is above chance level of p <0.5 (denoted by ^*^) for presence vs. absence of sound stimuli (p <0.05 Benjamini–Hochberg correction of the FDR for multiple comparisons, Matlab Multiple Testing Toolbox v1.1.0). ES = Effect size was measured using Hedge's g1 appropriate for use with one-sample t-tests (Matlab Toolbox: Measures of Effect Size). Values range from –inf to +inf with effect size considered as follows: small ±0.2, medium ±0.5, large ±0.8.

HR, hit rate, ratio of sound trials identified as sound trials; FAR, false alarm rate, ratio of sound absent trials identified as sound present trials; d′, dprime is a sensitivity index; SB, skateboarding conditions; Min, minimal cleaning; SE, Standard error; CI, 95% confidence interval.

To compare the classification performance of the various artifact cleaning pipelines a series of paired *t*-tests were conducted across the 20-fold cross-validation test set results. Separate series of analyses were carried out for rest (Table 3) and skateboarding (Table 4) conditions. The series of paired *t*-test comparisons are arranged such that if the value is positive, it means the column pipeline has greater performance and if the value is negative, it means the row pipeline has greater performance. The Benjamnimi–Hochberg correction of the FDR (*p* < 0.05) for multiple comparisons was used (Matlab Multiple Testing Toolbox v1.1.0). The effect size was measured using Hedge′s g that is appropriate for use with paired *t*-tests (Matlab Toolbox: Measures of Effect Size). Values range from –inf to +inf with effect size considered as follows: small ±0.2, medium ±0.5, large ±0.8.

**Table 3:** Rest condition.

	**Rest ASR**	**Rest ICA**	**Rest ICAASR**	**Rest ASRICA**
**Subject 1**				
Rest • Min	−2.29^*^ (−0.25)	−4.69^*^ (−1.37)	−4.27^*^ (−1.33)	−2.82^*^ (−0.71)
Rest • ASR		−3.93^*^ (−1.05)	−3.48^*^ (−1.02)	−1.41 (−0.41)
Rest • ICA			1.00 (0.05)	6.19^*^ (0.78)
Rest • ICAACR				5.79^*^ (0.74)
**Subject 2**				
Rest • Min	0.16 (0.01)	7.24^*^ (1.56)	7.24^*^ (1.56)	12.12^*^ (3.35)
Rest • ASR		7.30^*^ (1.60)	7.30^*^ (1.60)	12.90^*^ (3.45)
Rest • ICA			NaN	7.24^*^ (1.69)
Rest • ICAACR				7.24^*^ (1.69)
**Subject 3**				
Rest • Min	−0.90 (−0.06)	−1.83 (−0.47)	−1.38 (−0.36)	3.28^*^ (0.67)
Rest • ASR		−1.61 (−0.41)	−1.19 (−0.30)	3.69^*^ (0.74)
Rest • ICA			1.60 (0.10)	5.73^*^ (1.17)
Rest • ICAACR				4.76^*^ (1.03)

The paired t-tests are the columns – the rows (a negative value means the row is greater).

The T score is given on top and Hedge's g (effect size: small ±0.2, medium ±0.5, large ±0.8) is given in parentheses below (Matlab Toolbox: Measures of Effect Size).

^*^Denotes significant difference of the paired t-test for the various rest pipeline conditions across the 20 folds (p <0.05 Benjamini–Hochberg correction of the FDR for multiple comparisons, Matlab Multiple Testing Toolbox v1.1.0).

Min, minimal cleaning.

**Table 4:** Skateboarding condition.

	**SB ASR**	**SB ICA**	**SB ICAASR**	**SB ASRICA**
**Subject 1**				
SB • Min	14.33^*^ (3.96)	13.70^*^ (3.92)	13.30^*^ (3.81)	15.97^*^ (4.32)
SB • ASR		−0.71 (−0.21)	−0.83 (−0.24)	1.92 (0.47)
SB • ICA			−1.00 (−0.04)	2.74^*^ (0.69)
SB ICAACR				2.87^*^ (0.71)
**Subject 2**				
SB • Min	1.96 (0.44)	11.08^*^ (2.49)	6.84^*^ (1.81)	16.50^*^ (5.03)
SB • ASR		7.34^*^ (1.72)	3.69^*^ (1.09)	12.45^*^ (3.55)
SB • ICA			−4.80^*^ (−0.80)	6.42^*^ (1.90)
SB ICAACR				10.26^*^ (3.07)
**Subject 3**				
SB • Min	9.15^*^ (2.71)	18.28^*^ (5.57)	18.57^*^ (5.62)	16.57^*^ (5.05)
SB • ASR		7.76^*^ (1.93)	8.10^*^ (1.98)	5.25^*^ (1.70)
SB • ICA			1.00 (0.05)	−0.95 (−0.19)
SB • ICAASR				−1.12 (−0.24)

The paired t-tests are the columns – the rows (a negative value means the row is greater).

The T score is given on top and Hedge's g (effect size: small ±0.2, medium ±0.5, large ±0.8) is given in parentheses below (Matlab Toolbox: Measures of Effect Size).

^*^Denotes significant difference of the paired t-test for the various skateboard pipeline conditions across the 20 folds (p <0.05 Benjamini–Hochberg correction of the FDR for multiple comparisons, Matlab Multiple Testing Toolbox v1.1.0).

SB, Skateboarding conditions; Min, minimal cleaning.

For the rest condition (Table 3) ASR alone did not improve classification performance over minimal filtering for any of the three subjects. For subject 1 minimal cleaning was significantly better than any of the other pipelines using ASR and ICA. Subject 2 showed significantly better performance for all the pipelines containing ICA over that of minimal cleaning. Subject 3 only showed significantly better performance for the ASRICA pipeline over minimal cleaning. For subjects 2 and 3 ASRICA had significantly greater classification performance than all other pipeline conditions. Subject 1 also showed greater performance for the ASRICA pipeline over the ICAASR and the ICA pipelines.

The paired contrasts for the skateboarding condition (Table 4) showed that pipelines that included ICA had significantly better performance than minimal cleaning for all three subjects. ASR alone was better than minimal cleaning for subjects 1 and 3. The ASRICA pipeline had significantly better performance than the ICA and ICAASR pipelines for subjects 1 and 2. There were no pipelines that had significantly better performance than the ASRICA pipeline for any of the subjects.

**Figure 2:**
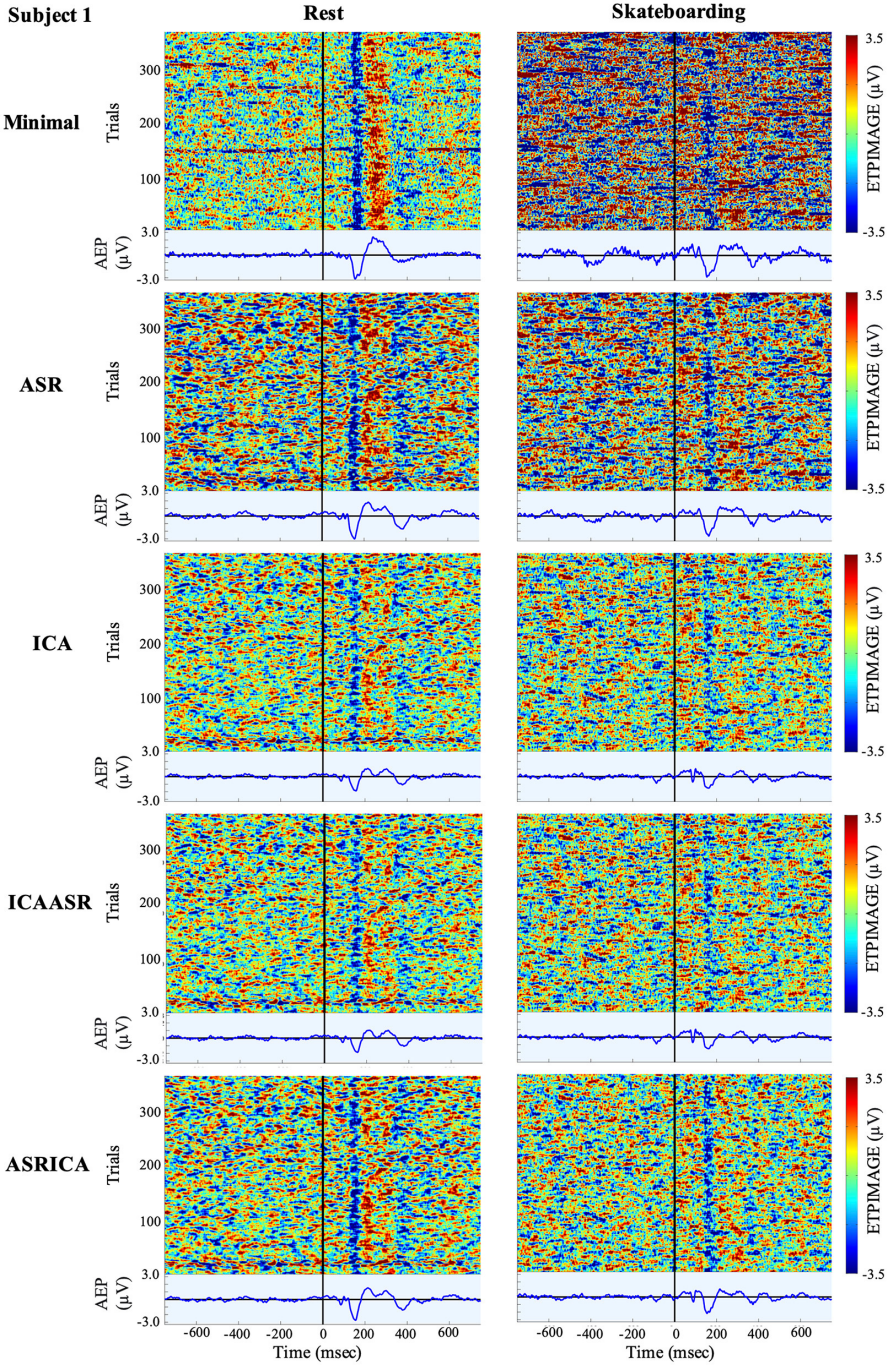
Event related potential (ERP) Image and auditory evoked potential (AEP) for Subject 1 at electrode Cz. The results for both the rest and skateboarding conditions for the five different cleaning pipelines are shown. The ERPImage displays the single trials on the y axis and time on the x-axis, with the color denoting the positive and negative amplitude. The AEP is the mean of the single trials displayed in the ERPImage. It should be noted that there is an ~45 ms constant delay in the Bluetooth delivery of the sound stimuli that has not been adjusted. The apparent lag in the timing of the N100 and P200 potentials are a result of this delay.

**Figure 3:**
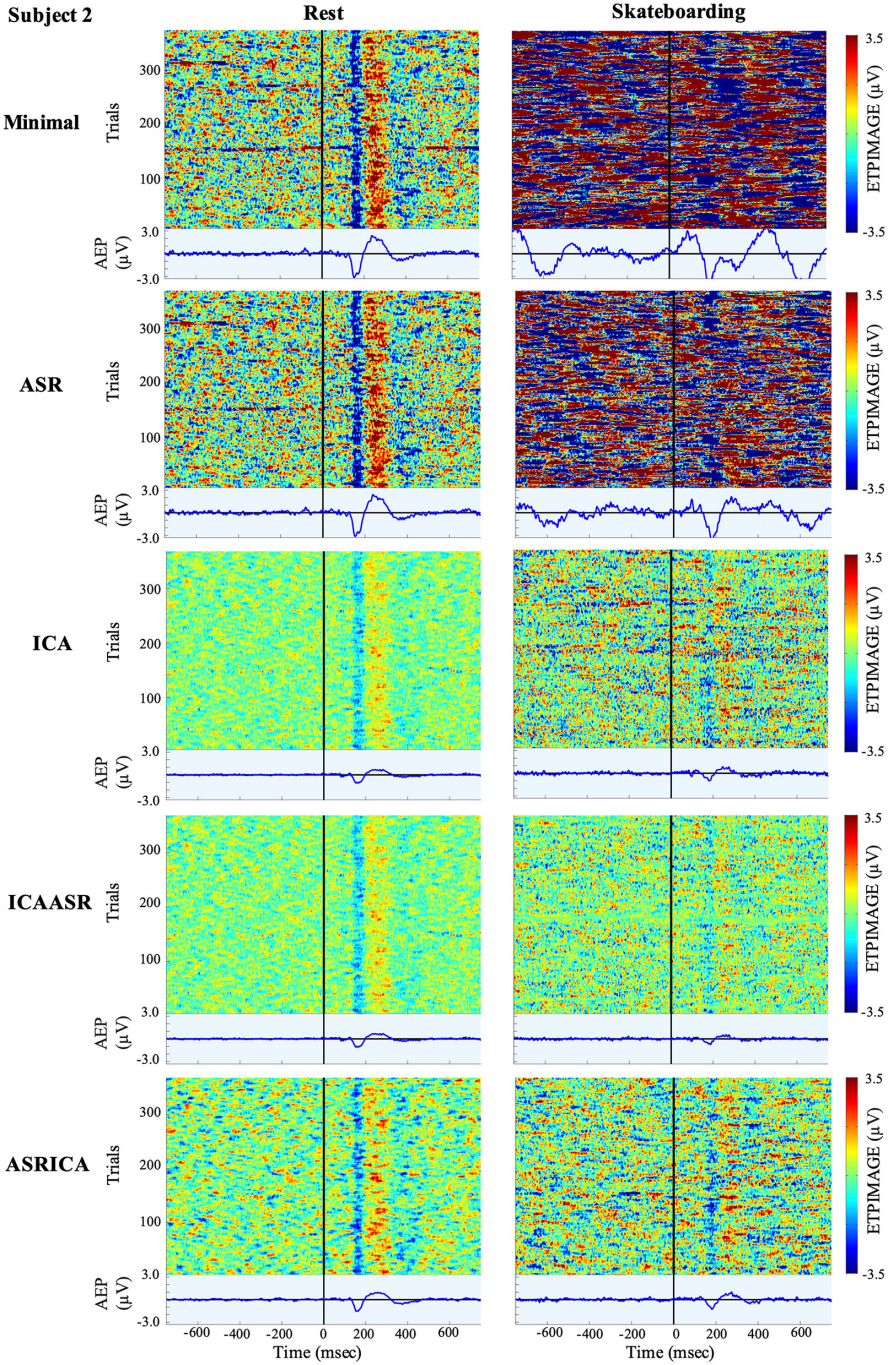
ERPImage and AEP for Subject 2 at electrode Cz.

**Figure 4:**
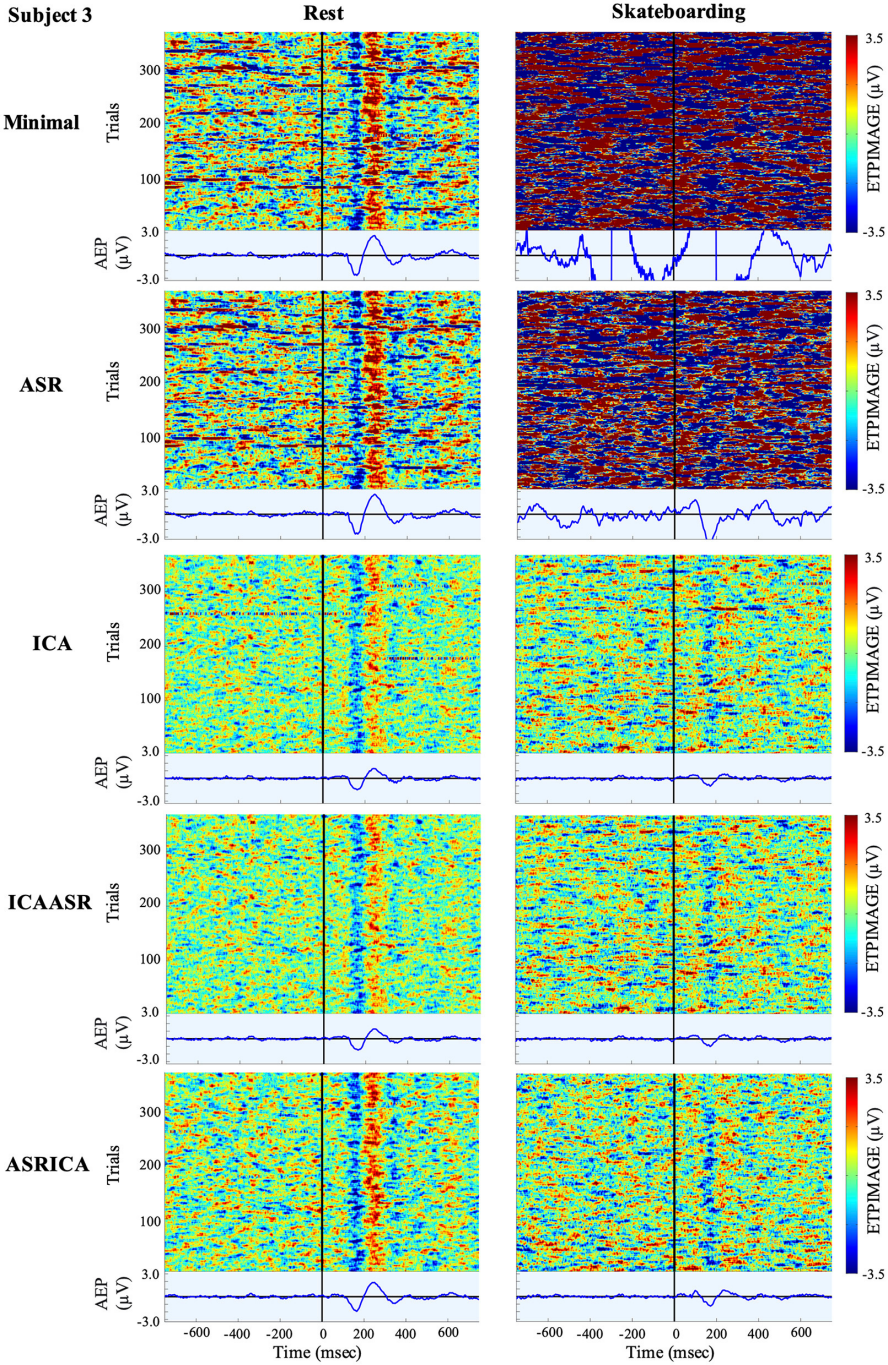
ERPImage and AEP for Subject 3 at electrode Cz.

Visualization of the effects of the various cleaning pipelines on the EEG for the rest and skateboarding conditions for all three subjects are shown using ERP Image (EEGLAB; see Figures 2–4). It can clearly be seen that the use of ICA and especially the combination of ASRICA has a profound effect of extracting the artifacts from the data over that of the minimal cleaning condition for the skateboarding condition both in observation of the single trials and the auditory evoked responses. For the rest condition the single trials and auditory evoked responses look fairly similar across the various cleaning pipelines. This is to be expected given the lower degree of artifacts in the resting condition relative to the skateboarding condition.

## Discussion

Although it was once thought impossible to record brain related EEG in real world sporting situations, the results of this experiment demonstrate that extraction of brain related activity and removal of artifacts resulting from skateboarding on a half-pipe ramp is possible by using techniques such as ASR and ICA. This demonstration is of general importance in further research involved with being able to investigate neural processes underlying motor gestures during sports. The classification analyses indicate that single trial performance at detecting presence and absence of an auditory stimuli is significantly improved by the use of ASRICA artifact cleaning over minimal artifact cleaning for the skateboarding condition for all three subjects (Tables 2, 4). The improvement in classification performance for ASRICA over minimal cleaning was 14.4%, 16.1%, and 13.3% respectively for the three subjects (all with enormous effect sizes). Similarly, *d*′ values increased for all three subjects for the skateboarding condition as a result of ASRICA cleaning over minimal cleaning (Table 2). It should be noted that all pipelines containing ICA performed significantly better than minimal cleaning for all three subjects for the skateboarding condition (Table 4). However, no other artifact cleaning pipeline performed significantly better than ASRICA for any of the subjects, and for two of the three subjects ASRICA was found to perform significantly better than ICA and ICAASR (with medium level effect sizes for one of the subject and enormous effect sizes for the other). The reason for the likely improvement of using ASR before ICA, rather than just ICA or using ASR after ICA, comes from the ability of ASR to clean non-stationary transient artifacts allowing for better ICA decomposition (Hsu et al., 2016; Chang et al., 2020). Evidence that using ASR before ICA results in better ICA decomposition is evident in the resulting number of IC brain components identified by ICLabel (Table 1). For all three subjects there was a substantial increase in the number of brain components (6, 9, and 3 respectively) for using ASR before ICA compared to ICA alone or using ASR after ICA (see Table 1).

In the Delorme (2023) study it was reported that minimal artifact cleaning using primarily high-pass filtering was better than most preprocessing pipelines tested for 3 experiments conducted in laboratory settings. While the combination of ASR and ICA did slightly better than minimal filtering in the Delorme (2023) study it was concluded that this improvement may be a confound of the channel removal and interpolation methods used rather than from ASR and ICA. Since all the pipelines we investigate in our study use the same channel removal and interpolation methods our results do not have this potential confound present in the Delorme (2023) study. The rest condition in our study is similar to the conditions present in laboratory settings. For two of the three subjects there was significant improvement in classification performance for ASRICA over minimal cleaning (Subject 2 = 12.3%; Subject 3 = 2.2%; see Tables 1, 3). However, for subject 1, the minimal cleaning pipeline had significantly better performance than all other pipelines including ASRICA (Table 3).

Visual inspection of the ERP Image (Taken at electrode Cz; Figures 2–4) clearly shows the extent of the massive artifacts imposed on the EEG signal during skateboarding with only minimal cleaning performed for all three subjects. For subject 1 (Figure 3) the negative potential (N100) time locked to auditory stimulus onset can be observed in the single trials to some extent, however, the AEP cannot be discerned over the considerable noise that occurs throughout the 1.5 s time course (both before and after auditory stimulus onset). For subjects 2 and 3 (Figures 3, 4) the artifacts resulting from skateboarding completely saturate the entire data with no clear pattern in the single trial data time locked to the onset of the audio stimulus. Furthermore, the AEP is not present for these subjects for the skateboarding condition with minimal artifact cleaning (Figures 3, 4). In contrast, for the rest condition with minimal cleaning the negative potential (N100) and the positive potential (P200) can be clearly observed in the single trial data as well as the AEP for all three subjects (Figures 2–4). Of considerable interest is that after ASRICA cleaning (as well as other pipelines using ICA) the ERP Image for the skateboarding condition for all three subjects (Figures 2–4) show observable negative potential (N100) and the positive potential (P200) in the single trial data as well as clear AEP. It is also apparently obvious in these figures for the skateboarding conditions that the background potential level in the data has been considerably reduced as a result of pipelines using ICA cleaning. It can also be observed from the ERP Image in the rest condition that the use of ASRICA considerably reduces the background artifacts across the entire time course (Figures 2–4). As a result of ASRICA (as well as other pipelines using ICA) cleaning the background potential levels of the ERP Image for the entire single trial time course are comparatively similar for the skateboarding and rest conditions for all three subjects (Figures 2–4). It can also be clearly observed that the single trial responses and the AEP are greater in magnitude and more apparent in the Rest than the Skateboarding condition. While, to some extent this difference may reflect an inability of ASRICA to remove all artifacts, there are also considerable additional factors that may be responsible. One difference is that the extra workload imposed by skateboarding likely reduces attention directed to the auditory stimuli. It is well known that single trial auditory (and visual) responses and AEPs (visual ERPs) are attenuated during dual task paradigms in which attention is toward a different task (Lavie, 2005; Molloy et al., 2015; Callan et al., 2018, 2023; Dehais et al., 2019; Ladouce et al., 2019). An additional reason why the single trial responses and the AEP are greater in magnitude and more apparent in the Rest than the Skateboarding condition may be a result of the acoustic masking of the auditory stimulus presented by earphone by the sound generated by the act of skateboarding on the ramp (mainly the contact and turning of the wheels of the skateboard on the ramp) that is not present during the resting condition.

There has been one previous EEG study conducted during skateboarding (Robles et al., 2021). In the Robles et al. (2021) study an electric skateboard was utilized to reduce the amount of muscle activity and body movement related artifacts. Their experiment involved a dual task paradigm in which the subject rode the electric skateboard around a 200 m oval track while listening to auditory stimuli. Attention related auditory evoked potentials could be detected in their experiment while in motion on the electric skateboard (Robles et al., 2021). Our study differs from that of Robles et al. (2021) in many ways. Our study focuses on the use of ASR and ICA as effective tools for cleaning artifacts from single trial (continuous) data while performing skateboarding maneuvers that require considerable whole-body movement and abrupt motion of the skateboard. In the Robles et al. (2021) study the electric skateboarding task was selected (going around an oval track) that reduced the degree of whole-body movement in order to reduce associated artifacts in the EEG data. Only minimal cleaning was utilized including band-pass filtering and regression techniques to remove eye movement and blinks from the EEG data. Instead of cleaning the EEG data, trials found to have excessive artifacts were completely removed from the analysis (Robles et al., 2021). This is in contrast to our study in which no trials were excluded from the analysis as a result of excessive artifacts. Rather, it was the goal of our experiment to remove the artifacts from the underlying brain related EEG by utilizing ASR and ICA. Our study further demonstrates that while minimal cleaning is not sufficient for skateboarding tasks requiring considerable motion (such as doing pumping and kickturns on a half-pipe ramp) with the use of ASR and ICA brain related EEG showing clear AEPs can be detected and classified at a single-trial level. Our results are consistent with other experiments conducted in real-world situations such as walking, running (Gorjan et al., 2022), playing the guitar, and piloting airplanes (Callan et al., 2015, 2018) in which ASR and ICA have been used to clean artifacts from the EEG data.

This is the first EEG study to look at skateboarding on a half-pipe ramp in which the skateboarder performs pumping and kick-turn maneuvers. The results of this experiment are important in that they strongly suggest that brain related activity can be successfully extracted from the EEG even in the considerably noisy situation of skateboarding on a half-pipe ramp by using ASRICA artifact cleaning. One potential confound may be that auditory stimuli that are presented during the flat part of skateboarding (where there are fewer movement related artifacts) may represent the only responses that were successfully classified. While we did not formally analyze the results in reference to this potential confound there are several reasons why this may not be problematic for our results. The time course of one single round trip on the half-pipe ramp is approximately as follows: flat (0.9 s), up and down the ramp (1.4 s), flat (0.9 s), up and down the ramp (1.4 s), for a total of about 4.6 s for the round trip. The movement and motion related artifacts are expected to be greatest on the ramp portion relative to the flat portion. The ramp portion takes up ~60% of the time and the flat part 40%. The duration of the single trials was 1.5 s divided into auditory present (750 ms after stimulus onset) and auditory absent (750 ms before stimulus onset) and randomly presented every 1.5–2 s. The position at which the auditory sound is presented will be randomly distributed but will in every trial likely include samples from the ramp portion where the signal is likely to have greater artifacts. If the ASRICA was not successful at cleaning these artifacts it is unlikely that the single trial classification performance for skateboarding would be more comparable to that of the rest condition than before cleaning with ASRICA. It should also be noted that the exact same trials are analyzed for the five artifact cleaning pipelines so their comparison is not confounded by the location on the ramp in which the auditory sound was presented.

## Conclusions

The auditory/skateboarding dual task paradigm used in this experiment demonstrates that brain activity can be extracted from EEG while doing whole body movements during real-world sporting activity (in this case skateboarding on a half-pipe ramp) using ASR and ICA artifact cleaning. EEG pre-processing pipelines including ASR and ICA were able to significantly clean artifacts in single trial data to a much greater extent than minimal pre-processing using primarily just filtering. These results strongly suggest that artifact cleaning using ASR and ICA could be used to investigate brain activity during various real-world situations in which there is considerable body movement, muscle activity, and motion related artifacts.

Future research can explore the extent to which modifying various ASR and ICA parameters may enhance cleaning performance in EEG data collected in real-world conditions with considerable movement and motion artifacts. The use of additional methods such as canonical correlation analysis CCA and different versions of ICA (AMICA, ORICA) as suggested by Gorjan et al. (2022) may also lead to enhanced artifact cleaning performance. With regards to our skateboard ramp research, now that cleaning of EEG data has been validated using ASR and ICA, the next step to be conducted in a subsequent experiment is to determine the brain activity related to transition to and initiation of specific whole-body gestures utilized for the skateboarding maneuvers (pumping and kick-turns) and ensure the results are not confounded by artifacts resulting from execution of the skateboarding maneuver itself.

The proposed artifact cleaning methods using expensive laboratory grade EEG equipment with a large number of channels (72 in this case) may limit its widespread use for general neuroergonomic applications. However, it is unlikely that artifact removal techniques using low-cost EEG units with few channels will be sufficient to adequately clean the data to be able to extract relevant brain activity under athletic like tasks in which there is considerable body movement. Professional training facilities and organizations as well as research institutes interested in understanding and enhancing neural processes underlying elite athletic performance would certainly be able to afford laboratory grade EEG equipment necessary for the proposed artifact cleaning methods to be employed.

It is often necessary to be able to record brain activity in the natural setting, not just in the laboratory or specialized training facilities. Many of the new laboratory grade EEG systems (with 64 or more channels) are extremely portable and can readily be used in real-world situations (Wascher et al., 2023). As have been demonstrated in use in airplanes in flight (Callan et al., 2015, 2018). In real-world environments that consist of considerable motion and movement related artifacts a combination of ASR and ICA will facilitate cleaning and extraction of brain related activity. It is generally recommended to have a greater number of channels (≥64 for high intensity movement) as it is related to the number of independent brain and artifact components that can be decomposed (Klug and Gramann, 2021; Gorjan et al., 2022). Additionally, if one is interested in source localization of brain activity it is also recommended to use high density (≥64 channels) especially in real-world situations (Klug and Gramann, 2021; Gorjan et al., 2022). One drawback of utilizing ICA for artifact cleaning in real-world settings is that it takes a considerable amount of processing time, such that, utilization of brain-computer-interfaces to give real-time feedback and/or manipulate devices is extremely challenging. However, it may be possible to use pretrained ICA weights, and/or utilize different versions of ICA (ORICA: Online Recursive Independent Component Analysis) (Hsu et al., 2016) that can be used with brain-computer-interfaces for real-time neuroergonomic applications. While one aspect of neuroergonomics focuses on the application of brain-based technology to improve human factors in real-world situations, it is also important to also realize that an additional goal of neuroergonomics is to understand how the brain functions in real-world situations such as while participating in athletics or other physically demanding activities in which real-time feedback and/or processing of the data is not necessary.

## Data availability statement

The raw data supporting the conclusions of this article will be made available by the authors, without undue reservation.

## Ethics statement

The studies involving humans were approved by ATR Human Subject Review Committee (ethics approval number 158). The studies were conducted in accordance with the local legislation and institutional requirements. The participants provided their written informed consent to participate in this study. Written informed consent was obtained from the individual(s) for the publication of any potentially identifiable images or data included in this article.

## Author contributions

DC: Conceptualization, Data curation, Investigation, Methodology, Project administration, Resources, Software, Supervision, Validation, Visualization, Writing – original draft, Writing – review & editing. JT-T: Data curation, Formal analysis, Investigation, Methodology, Software, Validation, Writing – original draft, Writing – review & editing. JL: Data curation, Investigation, Methodology, Software, Writing – review & editing, Writing – original draft. SI: Funding acquisition, Project administration, Resources, Writing – review & editing, Writing – original draft.
